# Clinical and satisfaction outcomes of using one or two dental implants for mandibular overdentures: preliminary short-term follow-up of a randomized clinical trial

**DOI:** 10.1186/s40729-020-00286-8

**Published:** 2021-02-11

**Authors:** Kássia Estefania Hauck, Micheline Sandini Trentin, Tarcio Hiroshi Ishimine Skiba, Jamil Awad Shibli, João Paulo De Carli

**Affiliations:** 1 University of Passo Fundo, Passo Fundo, RS Brazil; 2 School of Dentistry of the University of Passo Fundo, Rua Silva Jardim, 391-1301, Passo Fundo, RS 99010-240 Brazil; 3 University of Guarulhos, Guarulhos, SP Brazil; 4 Department of Periodontology and Oral Implantology, Dental Research Division, University of Guarulhos, Guarulhos, SP Brazil

**Keywords:** Implants, Overdentures, Satisfaction questionnaire

## Abstract

**Objective:**

This randomized clinical trial aimed to evaluate the marginal bone loss and peri-implant aspects in patients with mandibular overdentures retained by one or two implants and assess patient satisfaction, prosthesis-related clinical outcomes, and masticatory efficiency.

**Methods:**

Patients from the School of Dentistry of the University of Passo Fundo (UPF) with lower conventional complete dentures dissatisfied with prosthetic retention were selected. Eighteen patients were analyzed and divided into randomized treatment groups: GA, installation of one implant in the midline of the mandibular symphysis (8 patients), and GB, installation of two implants in the lower canine region (10 patients). Implant survival and prosthetic maintenance were assessed by clinical and radiographic examinations performed 6 months after implant placement and 3 months after loading. Finally, the masticatory efficiency of the prostheses was evaluated with the QoLIP-10 (Quality of Life with Implant-Prostheses) questionnaire, and the degree of patient satisfaction used the visual analog scale (VAS).

**Results:**

Regarding the esthetic satisfaction of the patients, there was no statistical difference between the two groups studied (*p* = 0.680). Patients who received two implants presented easier chewing (*p* = 0.049) and a lower average number of prosthesis maintenance. There was no difference between the groups regarding peri-implant bone resorption 3 months after the use of prostheses.

**Conclusions:**

The use of two dental implants showed higher masticatory ability and lower need for maintenance appointments when compared with one implant in mandibular overdentures but did not affect peri-implant aspects and patient satisfaction. The treatment using one implant was effective for the aspects evaluated, but further clinical studies are required on the subject.

## Introduction

More than 30% of the world population older than 60 years suffers from complete edentulism [1]. Progressive alveolar bone loss in elderly people with lower conventional complete dentures harms the quality of life, considering this type of rehabilitation does not present adequate retention and stability, also harming the masticatory ability and nutrition of these patients [2].

The oral rehabilitation most commonly performed in edentulous patients is the mucosa-supported complete denture. Although most individuals rehabilitated with conventional complete dentures are satisfied, a significant portion complains about esthetics, retention, and function, especially regarding the mandibular arch [3–5].

An alternative to improve retention, stability, and consequently functional aspects of complete mandibular dentures is using osseointegrated implants and the subsequent installation of overdentures [6]. The use of two dental implants for mandibular overdentures is already well established in the literature [7–10].

A cost/comparison study between mandibular overdentures retained by two dental implants and conventional complete mandibular dentures showed that the cost of an overdenture is 2.4 times the cost of conventional complete dentures [11]. It would be desirable for clinicians to offer a significant functional improvement over mandibular prostheses at an appropriate cost-benefit ratio, so the possibility of using one instead of two dental implants could reduce the cost significantly.

Defining the number of implants required to sustain a mandibular overdenture in patients with severe mandibular atrophy is still a controversial topic in the literature [12–15]. Mandibular overdentures retained by a single implant located in the mandibular symphysis have been proposed as an alternative treatment for elderly patients with prior experience of discomfort and functional difficulties with conventional full dentures [12–15]. There is some evidence that shows equivalent results with single implants when compared with two dental implants [16–18], but there is still a lack of indications for it, especially randomized clinical trials.

Considering that the success of this treatment modality, although remarkable, is beyond the purchasing power of many edentulous individuals, this randomized clinical trial aimed to evaluate the outcomes of the treatment with one or two dental implants for mandibular overdentures regarding satisfaction, marginal bone loss, and peri-implant aspects. The hypothesis is that mandibular overdentures supported by a single implant are as effective as mandibular overdentures supported by two implants.

## Methods

The Human Research Ethics Committee of the University of Passo Fundo approved this study (no. 2.572.556), which was registered in the Brazilian Clinical Registry Platform under number: RBR-4pt6wp.

All patients signed an informed consent form to participate in the research. The sample size was based on the Policastro VB et al. [19] study. The initial study included 20 patients with double conventional complete dentures (both jaws) who were dissatisfied with mandibular denture retention and stability and sought oral rehabilitation with mandibular overdentures at the School of Dentistry of the University of Passo Fundo (UPF), RS, Brazil.

Only the patients whose double full dentures were in good condition but with the lower prosthesis lacking adequate retention were eligible for the study. In such patients, implants were installed in the mandibular arch with subsequent transformation of conventional dentures into overdentures by capturing ball O-rings. Thus, for this parallel randomized clinical trial, only 18 patients were considered eligible and randomly assigned into two groups:

- Group A: installation of one implant in the mandibular symphysis region (GA—*n* = 8);
- Group B: installation of two implants in the mandibular canine area (GB—*n* = 10).

**Fig. 1:**
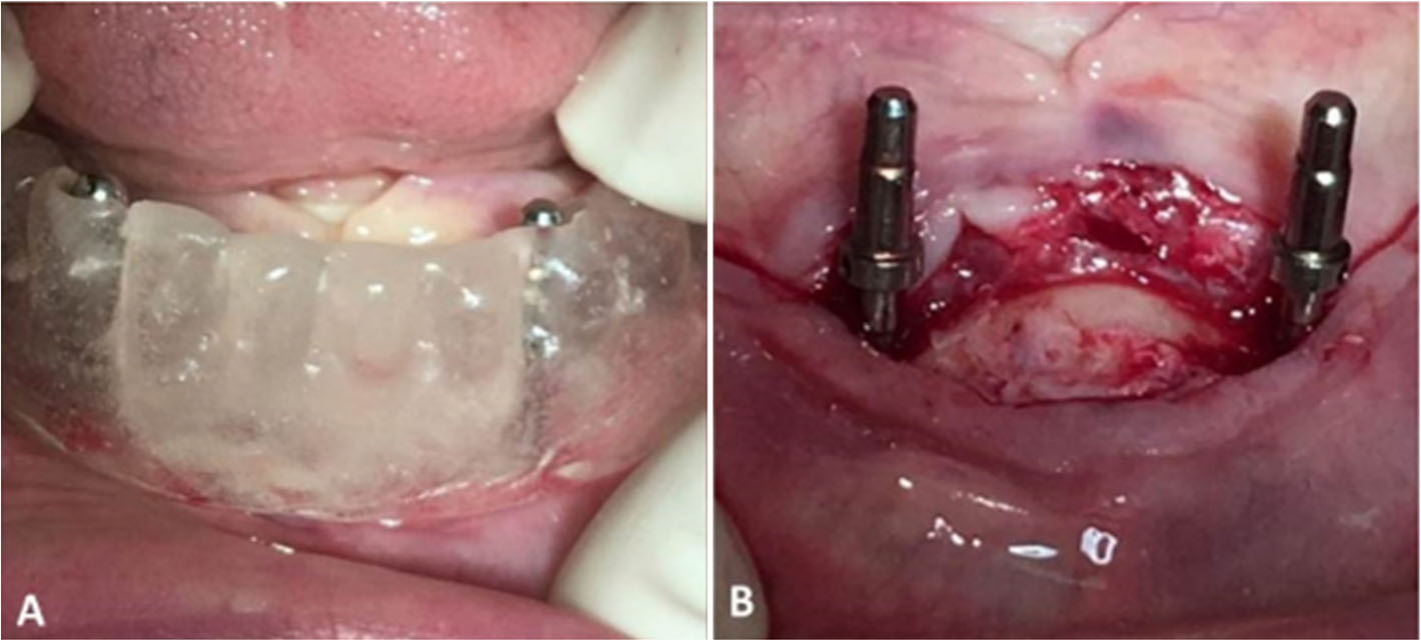
**a** Surgical guide positioned in the oral cavity during implant installation. **b** Parallelometers indicating parallelism in case of overdentures retained by two implants

Randomization was performed by a draw, in which the patient drew a number from an envelope (no. 1 for a single implant and no. 2 for two implants) and was subsequently referred to the particular type of surgery. A first researcher generated the random allocation sequence, a second researcher enrolled the participants, and a third researcher assigned participants to interventions (Fig. 1).

In all cases, the patients already had new mandibular complete dentures (performed by the Dental Prosthesis Laboratory of the School of Dentistry-UPF). The patients were evaluated to verify whether they were esthetically and functionally acceptable to continue the study. In addition to the physical examination and conventional prostheses, the screening process consisted of questions about the systemic health of patients and the request for a panoramic radiograph, which was used to verify bone height in the region to be rehabilitated with implants. Before implant surgery, complementary laboratory tests were requested (complete blood count, coagulogram, fasting glucose test, calcium, vitamin D, phosphorus, and alkaline phosphatase).

### Surgical procedure

**Fig. 2:**
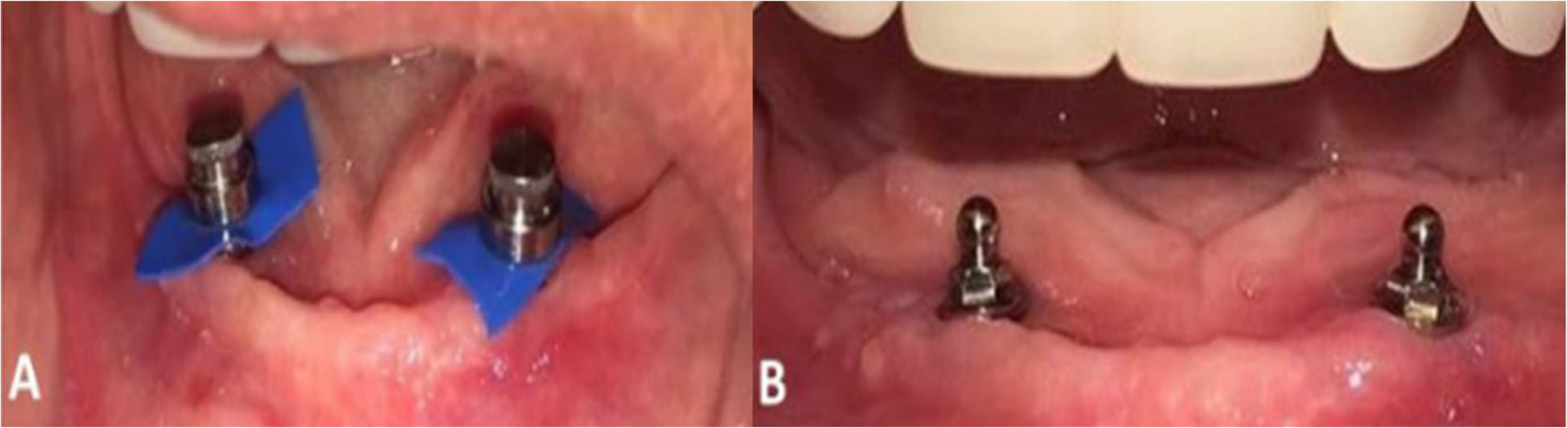
**a** O-rings: positioning of the capsules on the O-rings. **b** O-ring abutments after capturing the capsules at the base of the prosthesis

Two implant dentistry specialists installed the implant in an operating room of the university. Before installing the implants in each case, the old complete dentures of the patients were duplicated and a surgical guide was produced in colorless and self-curing acrylic resin (Jet™, Classic Dental Articles, São Paulo, SP, Brazil) (Fig. 2).

A total of 28 Morse taper implants with 4.1 mm of diameter and 9 to 11 mm of height (Singular, Parnamirim, RN, Brazil) were placed. Antibiotic prophylaxis (amoxicillin 1 g, 1 h before the procedure) was prescribed to selected patients.

Before the procedure, patients were anesthetized with 4% articaine. An envelope-like incision over the alveolar ridge crest was performed for better mucoperiosteum detachment and visualization of the surgical field. The bone was perforated with drills (Singular, Parnamirim, RN, Brazil). After installation, a torque of at least 32 Ncm was verified, with the platform installed at the bone level. The suture was performed with mattress-type stitches in the implant region and simple suture in the rest of the tissue surrounding the ridge. The postoperative medication continued with amoxicillin 500 mg (1 capsule every 8 h for 7 days), nimesulide 100 mg (1 pill every 12 h for 5 days), and paracetamol 750 mg (1 pill every 8 h for 3 days). The stitches were removed 10 days after the surgical procedure. There were no surgical complications observed in this study.

### Prosthetic procedure (capture of O-ring abutment capsules)

**Fig. 3:**
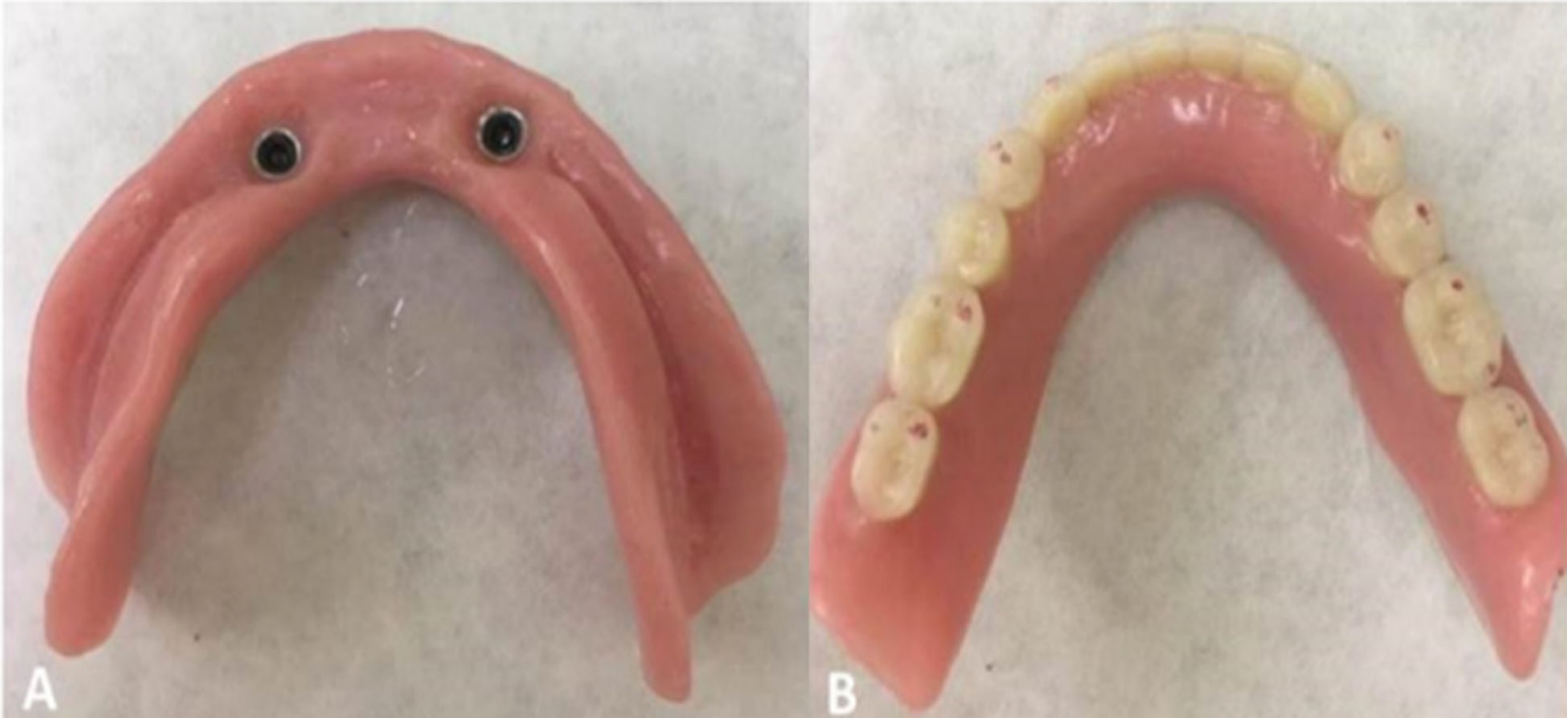
Capsule 1 and 2 implants. Capsules captured in the internal portion of the complete prosthesis

Approximately 12 weeks after the implant installation surgery, the O-ring abutment capsules (Singular, Parnamirim, RN, Brazil) were captured. This procedure was performed by positioning the capsule on the abutment and conducting an internal relief of the prosthesis in the region of the implant(s) (Fig. 3a). After placing the self-curing acrylic resin inside the prosthesis, it was placed in the mouth with the patient in a centric position for 5 min. A metal infrastructure was not used in lower dentures.

An occlusal adjustment was also performed at this stage. The patient was instructed regarding the care with prosthesis placement and removal, as well as oral hygiene in the region of the implants and prosthesis hygiene.

### Outcomes analyzed and evaluation period

The patients treated in this study were evaluated for the following outcomes: peri-implant bone loss, peri-implant tissue aspects, prosthesis maintenance episodes, and implant/prosthetic satisfaction.

The implant/prosthetic satisfaction questionnaire, bone loss assessment, and analysis of tissue aspects were performed with the patients 3 months after implant prosthesis installation (implant loading). Maintenance episodes were analyzed and cataloged according to the needs of each patient, as well as cases of fractures or prosthesis adjustments.

### Peri-implant tissue aspects

The peri-implant condition was assessed 3 months after implant loading. The following parameters were analyzed:

- Distance of the gingival margin to the prosthetic platform: this distance was measured using a graduated periodontal probe at the buccal, lingual, mesial, and distal interfaces for 1 or 2 implant cases. If the implant platform was above the gingival margin, a negative value was attributed to the measurement found;
- Presence of bleeding on probing: a periodontal probe was used to observe the occurrence or not of bleeding on probing on the four aspects of the implant (buccal, lingual, mesial, and distal);
- Presence of biofilm: observed on the O-ring surface and recorded as “present” or “absent,” according to Silness and Loe [20].

### Peri-implant bone loss

Peri-implant bone loss was assessed by a periapical radiograph taken with a radiographic positioner 3 months after implant loading (6 months after implant placement surgery) and analyzed using the Image Tool software to determine whether the bone loss occurred in the peri-implant region. Bone losses related to the implant platform (reference point) and bone tissue in the mesial and distal areas were measured (mm).

**Fig. 4:**
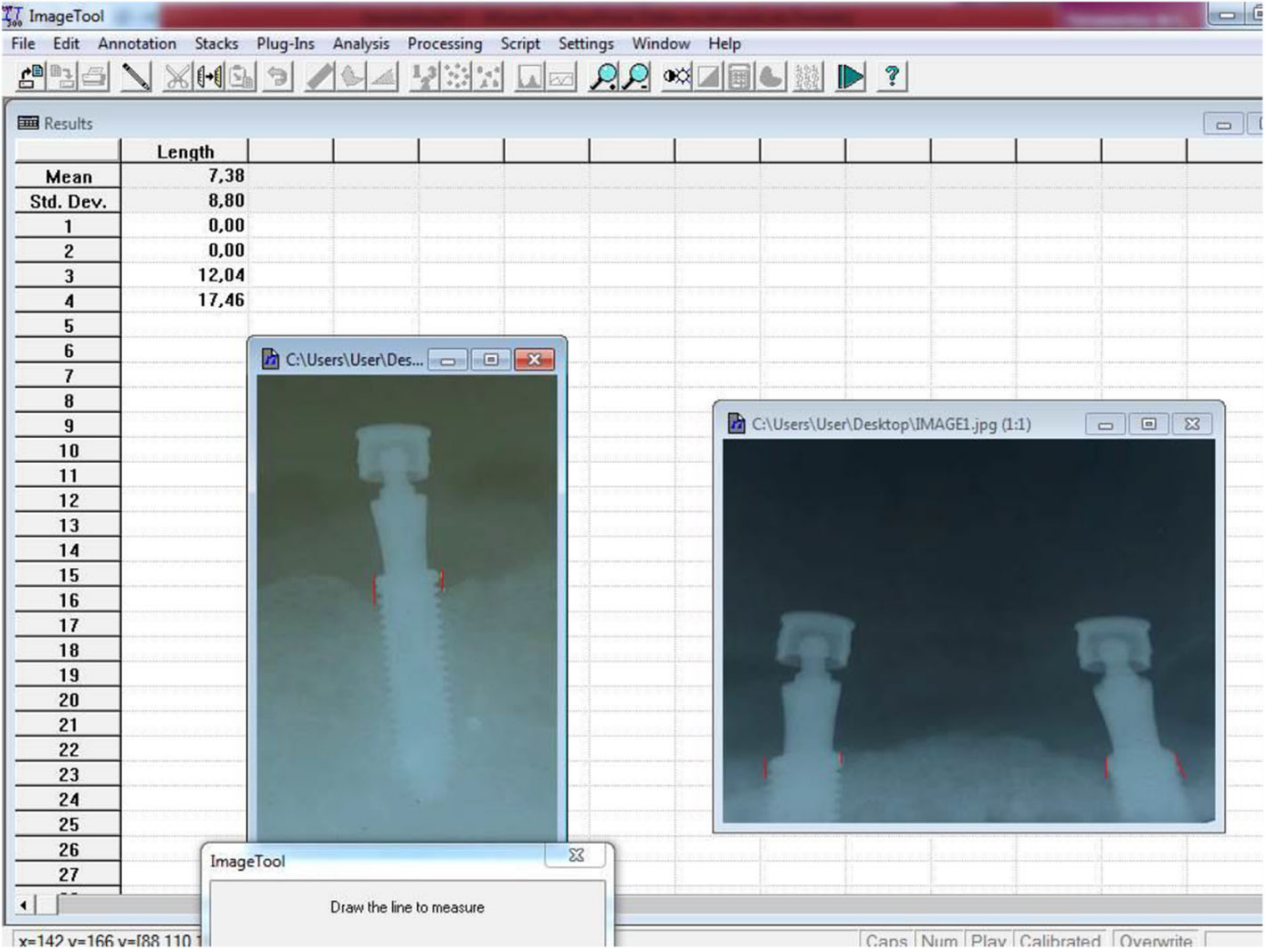
Measurements in millimeters of bone resorption implant platform and bone crest

**Fig. 5:**
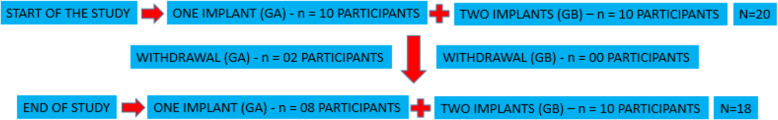
Explanatory flowchart of the study sample

Having determined a reference point common to all implants, defined as the implant platform, bone resorption was calculated by measuring the distance from the marginal bone to the level of the implant platform (Fig. 4). The measurements were performed by two calibrated dental surgeons, in which the final average of the two measurements performed on each side (mesial and distal) was used. These bone levels were measured in millimeters (Fig. 5). The implants were classified as follows: (1) with unilateral bone loss (mesial or distal), (2) with bilateral bone loss (mesial or distal), and no bone loss [21].

### Maintenance episodes

The number of times needed for prosthesis maintenance during the follow-up period of the mandibular overdentures in the study participants was recorded. Maintenance included occlusal adjustment and potential discomfort with the prosthesis in regions of the base presenting inadequate adaptation and fractures. Maintenance was recorded 3 months after the end of treatment (implant activation).

### Evaluation and questionnaire of implant/prosthetic satisfaction

The main instrument for comparing patient satisfaction in the groups studied with overdentures was the QoLIP-10 questionnaire, considering it is validated [22] and worked as an instrument for evaluating implant on prostheses in previous studies [23, 24]. Implant/prosthetic satisfaction was assessed and the patients were classified as “satisfied,” “normal” (nothing changed), or “dissatisfied” or “yes” and “no,” depending on the question.

### Data analysis

After data collection, the results were tabulated using the Excel™ software and statistically analyzed using the Statistical Package for Social Science 19.0™ (SPSS). At this stage, descriptive analysis and non-parametric tests were performed to evaluate the association between the groups defined with the variables studied. The statistic test used was the Mann-Whitney, at 95% confidence intervals, considering *p* < 0.05.

## Results

Figure 5 describes in detail the sample of each group at the beginning and end of the study. This study started in April 2017, extending until July 2019, and it was finished because of the pandemic. Two participants in GA (one implant) withdrew because they reported discomfort during the research.

Table 1 describes the sample studied in the present research.

**Table 1:** Inclusion and exclusion criteria of patients

Sample			Total
Sex (total sample)	11 (61.11%) women	7 (38.89%) men	18 (100%)
Age (total sample)	51 to 76 years old	− average of 64.78	S.D.± 7.64
One implant (GA)	4 (50%) women	4 (50%) men	8 (100%)
Two implants (GB)	7 (70%) women	3 (30%) men	10 (100%)
Total of implants	18 (64.29%) women	10 (35.71%) men	28 (100%)

In this test, only chewing difficulty was statistically different for the study groups and it was the least favorable result for the group with only one implant installed (GA). The other items of the questionnaire did not present significant differences between the groups, as seen in the analysis of the QoLip-10 questionnaire (Table 2).

**Table 2:** Analysis of the QoLip-10 questionnaire

	One implant (GA) ***n*** (%)	Two implants (GB) ***n*** (%)	***p*** value*
**Esthetic satisfaction**			0.680
Satisfied	7 (87.5)	8 (80.0)	
Normal	1 (12.5)	2 (20.0)	
Dissatisfied	0 (0.00)	0 (0.00)	
**Satisfaction with chewing**			0.227
Satisfied	3 (37.5)	6 (60.0)	
Normal	2 (25.0)	3 (30.0)	
Dissatisfied	3 (37.5)	1 (10.0)	
**Satisfaction with the prosthesis**			0.135
Satisfied	4 (50.0)	8 (80.0)	
Normal	2 (25.0)	2 (20.0)	
Dissatisfied	2 (25.0)	0 (0.00)	
**Oral pain**			0.208
No	1 (12.5)	4 (40.0)	
Yes	7 (87.5)	6 (60.0)	
**Speaking difficulty**			0.805
No	6 (75.0)	8 (80.0)	
Yes	2 (25.0)	2 (20.0)	
**Chewing difficulty**			0.049
No	0 (0.00)	4 (40.0)	
Yes	8 (100.0)	6 (60.0)	
**Difficulty in oral hygiene**			0.264
No	7 (87.5)	10 (100.0)	
Yes	1 (12.5)	0 (0.00)	
**Concern**			0.401
No	4 (50.0)	7 (70.0)	
Yes	4 (50.0)	3 (30.0)	
**Communication difficulty**			0.410
No	6 (75.0)	9 (90.0)	
Yes	2 (25.0)	1 (10.0)	
**Difficulty in daily activities**			0.264
No	7 (87.5)	10 (100.0)	
Yes	1 (12.5)	0 (0.00)	

All patients needed prosthetic maintenance, and those who had only one implant installed required more maintenance appointments (Table 3).

**Table 3:** Number of maintenances with one and two implants

	***N*** (subjects)	Minimum	Maximum	Average	S.D.
**General maintenance**	18	1	5	2.78	1.309
**Two implants**	10	1	5	2.20	1.135
**One implant**	8	2	5	3.50	1.190

The complications associated with prosthesis and implants were low, showing only one prosthesis fracture, no implant loss or suppuration, and low plaque and bleeding indexes (Table 4).

**Table 4:** Results related to the outcomes found in prostheses and implants

	*N*	*N*	%	%
	One implant	Two implants	One implant (%)	Two implants (%)
**Prosthesis fractured**	1	0	12.5	0
**Implant loss**	0	0	0	0
**Suppuration**	0	0	0	0
**Plaque index**	7	8	87.5	80
**Bleeding index**	5	2	50	25.5

The data for peri-implant bone loss and probing depth were collected in the same period, respectively, and no statistical difference of marginal bone loss was found between the groups (Table 5).

**Table 5:** Peri-implant bone remodeling (mm)

		$\overline{\boldsymbol{x}}\pm \boldsymbol{s}$	***p*** value*
**One implant**	**Distal**	1.08 ± 0.87	0.8560
	**Mesial**	1.01 ± 0.61	
**Two implants**	**Distal**	1.76 ± 1.12	0.2620
	**Mesial**	1.24 ± 0.79	
**Total bone loss**	**One implant**	1.04 ± 0.69	0.2420
	**Two implants**	1.50 ± 0.98	
**Group**	**Average (mm)**		
	Mean	SD	*p* value
**1 implant**	1.04	(0.69)	0.232
**2 implants**	1.42	(0.84)	

## Discussion

Patient reassessments occurred 3 months after implant activation. There were no statistical differences between esthetic satisfaction and prosthesis satisfaction in both groups. Nevertheless, when assessing satisfaction with masticatory aspects, more individuals were satisfied in group B (two implants) than in group A (one implant). Thus, except for masticatory function, both patients with one and two implants were satisfied with the treatments performed. These results add to the findings by Patil and Seow [21], who analyzed 24 patients divided into two groups (one and two mandibular implants) and concluded there were no differences between the maintenance of alveolar bone height and the satisfaction of both groups after 1 year. In the present study, initially, 10 participants were chosen for each study group (group A and group B). However, in the course of the study, two participants in group A (a single implant) quit participating in the evaluations.

In the present study, in addition to 1 g of amoxicillin administered 1 h before surgery, the postoperative medication continued with amoxicillin 500 mg (1 capsule every 8 h for 7 days), nimesulide 100 mg (1 pill every 12 h for 5 days), and paracetamol 750 mg (1 pill every 8 h for 3 days). There were no surgical complications observed in this study, and the pharmacological protocol was based on previous studies such as by Lang et al. [25] who conducted a systematic review in MEDLINE (PubMed) and the Cochrane Library from 1991 to July 2010 and stated that lower failure rates were found in groups provided with a course of postoperative antibiotics.

The results of this study also corroborate the findings of Ismail et al. [26]. In 2 years of follow-up, they evaluated 10 users of overdentures supported by a single implant who were satisfied with the stability, retention, and esthetics of the prosthesis. Similarly, in the study by Abou-Ayash *et al*. [27], 158 patients with mandibular overdentures supported by a single implant were evaluated through a randomized clinical trial comparing different implant loading protocols, and no differences were found regarding patient satisfaction.

Lee *et al*. [28] performed a systematic review aiming to evaluate the influence of the number of implants on the maintenance of overdentures, the remaining alveolar bone, and the patient’s degree of satisfaction. After evaluating 11 studies (out of 1098), the authors concluded that the number of implants did not affect negatively the physiological, functional, or psychological aspects of overdenture users. The authors also state overdenture users were much more satisfied after the treatment than before, regardless of the number of implants, which shows that treatment with overdentures improves the quality of life of completely edentulous patients. Such statements corroborate most of the findings of this study.

According to Mahookar *et al*. [29], the midline single implant system was approved as a cost-effective and successful treatment. However, clinical parameters such as masticatory efficiency, strength, retention, and stability still need to be investigated. These findings can be confirmed in this study because of the greater dissatisfaction with masticatory function and greater need for prosthetic maintenance in users of overdentures supported by a single implant when compared to users of prostheses supported by two implants. The other variables measured by the QoLIP-10 questionnaire (pain, phonation difficulty, oral hygiene difficulty, concern, communication difficulty, and difficulty in daily activities) showed no statistical difference between the groups studied.

In this study, the indexes of biofilm, gingival bleeding, and probing depth around the implants were similar in both groups. Such healthy gingival conditions are probably due to the adequate oral hygiene practiced by the patients of the sample, considering the gingival tissues around all implants studied showed signs of mild inflammation. Thus, it may be inferred that advanced age and reduced ability of elderly patients do not represent a higher risk for the development of peri-implant injuries [21]. This was confirmed by the results of this study, in which satisfactory peri-implant mucous parameters were compatible with tissues throughout the follow-up period.

Regarding the average number of maintenance appointments after the delivery of overdentures, group A (one implant) presented higher values than group B (two implants). This finding differs from the studies by Lee *et al*. [28] and Policastro et al. [19], who state that prosthesis maintenance and patient satisfaction are probably not affected directly by the number of implants. According to these authors, the most common type of prosthetic maintenance and complication is prosthetic repair due to the fracture that occurs around the O-ring of prostheses retained by a single implant. It is noteworthy that, in this study, an episode of this type of fracture occurred, precisely in group A.

The findings of this study also differ partially from the statements by Walton *et al*. [30], according to whom maintenance, component costs, and treatment time were lower in overdentures supported by one implant compared to overdentures supported by two implants. Three months after implant loading, a marginal bone loss of 1.04 ± 0.69 mm was observed in the 1-implant group and 1.50 ± 0.98 mm in the 2-implant group, with no statistical difference between them (*p* = 0.2420). This finding agrees with the study by Ismail *et al*. [26], according to which both groups (one or two implants) presented slight marginal bone loss. For the authors, radiographic examinations revealed that most of the marginal bone resorption occurred within the first 6 months of the prosthetic load. In this study, we evaluated peri-implant bone loss using standardized periapical radiographs in the Image Tool software, which is a possible limitation of this study.

Kronstrom et al. [18] found results similar to this study, as they evaluated patient satisfaction and clinical outcomes among individuals with mandibular overdentures supported by one or two implants, 5 years after loading. Thirty-six individuals (16 men and 20 women) received one or two implants in the anterior mandibular region. Seventeen individuals (seven men and 10 women) with an average age of 59.4 years (ranging from 44 to 74 years old) were followed-up for 5 years. No implant has failed between the 12- and 60-month follow-up exams, and the need for prosthesis maintenance was low. The mean peri-implant bone change was 0.92 mm. Patient satisfaction scores increased significantly compared to the baseline and remained high in both groups, with no significant differences. No significant differences were found among the individuals. Another limitation of our study is that assessing the quality of life of individuals through the OHRQoL index requires a longer follow-up. We emphasize that this study will continue to be developed and aims to perform the longitudinal monitoring of these patients for 5 years.

In this study, no implant losses were noted during the treatment period. Contrary to our findings, Bryant *et al*. [31] reported that none of the implants failed in the overdentures supported by one implant, but five implants failed in four participants of the 2-implant group, even before implant loading. Lee *et al*. [28] concluded in a systematic review that the survival rate of implants below mandibular overdentures is high, regardless of the number of implants. Trial follow-up ranged from one to 10 years, and implant survival rate in 10 of 11 trials ranged from 95 to 100% under conventional loading. Immediate loads showed lower implant survival rates. It is noteworthy that in this study, for reasons of standardization and safety, the conventional loading (late) of implants was chosen and there were no implant losses.

Ahmed Elawady *et al*. [32], in a systematic review with meta-analyses, analyzed peri-implant aspects and implant failures of mandibular overdentures with one and two implants including only five randomized controlled trials and showed that the single implant “was better than the 2-implant mandibular overdenture in terms of marginal bone loss and implant failure.”

In another systematic review with meta-analyses comparing the peri-implant aspects and patient satisfaction of one and two implant-retained overdentures, Alqutaibi *et al*. [33] included only three randomized controlled trials and such low number of studies was due to the “paucity of literature regarding randomized controlled trials on single implant overdentures.” Their results were also similar to the findings of this study, with no significant statistical difference between the groups for patient satisfaction and peri-implant aspects.

A possible explanation for the good results of one compared with two dental implants for mandibular overdenture was shown by Liu *et al*. [34] in a three-dimensional finite element analysis, in which, contrary to common belief, the stress value for axial and lateral forces generated on implant/abutment complex was lower in the abutment and bone around single implants. The reason is simple: in single-implants, the overdenture can rotate over the implant side to side without an increase of strain, which does not occur with two implants, because it only rotates on the fulcrum line through the implants, increasing the strain.

The limitations of this study include the short follow-up time, considering that 3 months is not sufficient to evaluate the effect of time on overdenture wear, the inability to blind the participants to the treatment modality, and the small sample of only 18 participants. Mandibular overdentures with two implants are mostly considered the first choice of treatment [7–10], although there is a lack of randomized controlled trials comparing one and two dental implants for overdentures. Despite all limitations, the present study is valuable for the literature and did not cause damage to the participants of both groups evaluated.

## Conclusion

It may be concluded that oral rehabilitations with overdentures supported by one or two implants were not different in terms of patient satisfaction and peri-implant aspects (bleeding, probing depth, and peri-implant bone loss). However, patients who received two implants showed higher masticatory ability and lower need for maintenance appointments when compared to the group of patients with prosthesis supported by one implant. The use of one or two implants did not affect patient satisfaction and peri-implant aspects. The treatment using a single implant was an effective and reliable alternative for mandibular overdentures, at least after a short period of evaluation.

## Data Availability

Not applicable.

## Abbreviations

IBGE: *Instituto Brasileiro de Geografia e Estatística* (Brazilian Institute of Geography and Statistics)
UPF: University of Passo Fundo
GA: Group A
GB: Group B
QoLIP-10: Quality of Life with Implant-Prostheses
SPSS: Statistical Package for Social Science
